# Traceability and Adulteration Analysis of Citri Reticulatae Pericarpium Based on “Digital Identity Card” and UHPLC‐QTOF‐MS Analysis

**DOI:** 10.1002/fsn3.70163

**Published:** 2025-04-30

**Authors:** Xianrui Wang, Jiating Zhang, Fangliang He, Wenguang Jing, Xiaohan Guo, Minghua Li, Kunzi Yu, Jianbo Yang, Xianlong Cheng, Feng Wei

**Affiliations:** ^1^ Institute for Control of Traditional Chinese Medicine and Ethnic Medicine National Institutes for Food and Drug Control Beijing China

**Keywords:** adulteration, Citri Reticulatae Pericarpium, digital identity card, matching credibility, traceability, UHPLC‐QTOF‐MS

## Abstract

In order to improve the identification efficiency, strengthen quality control, and realize the quick traceability and adulteration analysis of Citri Reticulatae Pericarpium (CRP) and Xinhui's Citri Reticulatae Pericarpium (XCRP, geoauthentic herb), UHPLC‐QTOF‐MS combined with “digital identity card” was used to explore digital identification of CRP. UHPLC‐QTOF‐MS chemical analysis was conducted on CRP from different origins, and after quantized processing, the shared ions were extracted from different batches of CRP from the same origin as the “ions representation” of CRP of the same origin. Further, the data set of unique ions of each origin relative to other origins was screened out as “digital identity card” of CRP from each origin. In addition, the CRPs that do not originated from Xinhui were mixed and prepared as reference samples. Then the reference samples and XCRP were used to extract “digital identity card” of CRP and XCRP again. Based on the matching of the above “digital identity card” and feedback matching credibility (MC) to realize the traceability and adulteration analysis of CRP. The results showed that the traceability analysis can be effectively realized based on the “digital identity card” of CRP from different origins, and the MC was as high as 90%. Even if 5% of CRP was added to XCRP, it can still be effectively detected in adulteration analysis. GRP's origin traceability and adulteration analysis can be realized based on UHPLC‐QTOF‐MS analysis and “digital identity card” effectively and accurately. It has important reference significance for the quick digital analysis of traceability and adulteration of CRPs and digital quality control of Chinese medicines.

## Graphical abstract

GRP's origin traceability and adulteration analysis can be effectively and accurately realized on the basis of UHPLC‐QTOF‐MS analysis and “digital identity card.” It has important reference significance for the quick digital analysis of traceability and adulteration of CRPs and digital quality control of Chinese medicines.

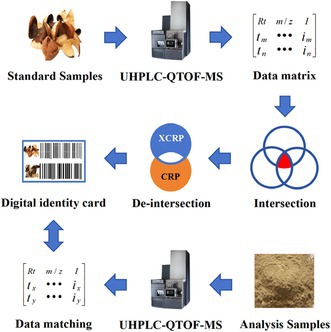

## 1 Introduction

Citri Reticulatae Pericarpium (CRP) is the dried and mature pericarp of *Rutaceae Citrus reticulata* Blanco and its cultivated varieties (Yu, Sun, et al. 2018). CRP has rich medicinal and edible value. CRP is widely produced in Hunan, Hubei, Guangxi, and other places in China; among them, the CRP produced in Xinhui (XCRP) is considered a geoauthentic herb that has a higher market price and is superior to other producing areas in clinical application and pharmacodynamic function (Zhang, Jiang, et al. 2024). However, the production of XCRP is limited, and the demand exceeds supply in the market, so some unscrupulous merchants take advantage of the similarity of CRP and XCRP in appearance to substitute the good for the bad and seek illegal interests. Therefore, it is necessary to strengthen the traceability analysis, market supervision, and quality control of XCRP and CRP.

To this end, relevant scholars have carried out many studies. For example, Zheng et al. conducted the identification of XCRP and CRP using UHPLC‐Q‐TOF‐MS/MS and multivariate statistical analysis. The results showed that XCRP and CRP could be effectively differentiated, and the potential biomarkers of XCRP were Rhoifolin and Diosmetin‐6‐C‐glucoside (Zheng et al. 2019). Wang et al. distinguished XCRP and CRP using UHPLC‐QTOF‐MS and support vector machine (SVM) techniques effectively. Furthermore, the results showed that components such as nobiletin and natsudaidin may also be potential chemical markers for distinguishing XCRP and CRP (Wang et al. 2019). Chen et al. conducted an adulteration analysis of XCRP and CRP using FT‐NIR and machine learning techniques and achieved a model accuracy as high as 98.64% (Chen et al. 2024). In addition, thin layer chromatography, high performance liquid chromatography, and DNA barcoding are also used for the traceability analysis of CRP (Zheng et al. 2018; Yu, Zhang, et al. 2018). All the above studies were helpful to the analysis of CRP; however, there were some shortcomings: (1) most current analyses focused on the discrimination of XCRP and CRP, rather than on adulteration identification. (2) Even if the traditional identification method can achieve adulteration identification, it is also time‐consuming and labour‐intensive. (3) On the other hand, the traditional identification method is based on only two or three chemical components, which is not representative and unconvincing, as well as it does not utilize the information of unknown components reasonably and effectively.

Given the above problems and shortcomings, and considering the arrival of the digital age, this study carried out the identification and adulteration analysis of XCRP and CRP based on the digital characterization of chemical components (Zhang, He, et al. 2024; Wang, Zhang, Jing, Guo, et al. 2024). Firstly, the UHPLC‐QTOF‐MS was utilized to analyze CRP and XCPR under the unified analysis conditions. Secondly, the Progenesis QI software (Version 2.4.69) was used to digitize the mass spectrometry of CRP and XCRP samples to obtain digital characterization of chemical components. Then, the shared ions were extracted from different batches of CRP from same origin as the “ions representation” of CRP of same origin (Wang, Zhang, He, Fu, et al. 2024). Further, the data collection of unique ions of each origin relative to other origins were screened out as “digital identity card” of CRP from each origin to realize traceability analysis (Rui Wang et al. 2024). In addition, the CRPs that do not originate from Xinhui were mixed and prepared as reference samples. Then the reference samples and XCRP samples were used to extract “digital identity card” of CRP and XCRP again for adulteration analysis (Wang, Zhang, Jing, Li, et al. 2024). The traceability and adulteration analysis of CRP were realized based on the matching of “digital identity card” and feedback matching credibility (MC).

## 2 Materials and Methods

### 2.1 Herbal Materials

33 batches of medicinal materials, including Hunan's CRP (6 batches), Hubei's CRP (6 batches), Guangxi's CRP (6 batches), Yunnan's CRP (6 batches), and Xinhui's CRP (9 batches) were collected by the National Institutes for Food and Drug Control. All samples were identified by the laboratory and met the requirements of the Chinese Pharmacopeia; the detailed information about materials was shown in Table S1. In addition, all the materials were stored in a cool and dry place.

### 2.2 Reagent Materials

Mass spectrometry‐grade methanol (Lot: ED341‐CN) was purchased from Honeywell Trading Co. Ltd. of Shanghai, China. Mass spectrometry‐grade acetonitrile (Lot: 222372) was purchased from Thermo Fisher Scientific Shier Technology Co. Ltd. of Shanghai, China. Mass spectrometry‐grade formic acid (L1670) was purchased from Honeywell Trading Co. Ltd. of Shanghai, China. Ultrapure water (GB 19298) was purchased from Watsons Food and Beverage Co. Ltd. of Guangzhou, China.

### 2.3 Sample Pretreatment

The raw materials were crushed into powder and filtered by No. 3 sieve. The CRP powder from different regions (excluding Xinhui) was mixed to make 8 reference samples for extracting the “digital identity card” of CRP. In addition, different proportions of CRP powder of reference samples was added to the XCRP powder (5%, 10%, 20%, 30%, 50%) to make mixed positive samples. Accurately weigh 1.00 g herbal material dried powder to place in 50 mL tapered bottle with a plug respectively; then accurately add 25.00 mL of methanol with a pipette into tapered bottle to perform ultrasound for 30 min (power: 500 W, frequency: 40 kHz); Finally taking out and cooling to room temperature, as well as filtering with 0.22 μm filter membrane to obtain samples. The samples were stored at 4°C in the refrigerator before UHPLC‐QTOF‐MS analysis.

### 2.4 UHPLC‐QTOF‐MS Analytical Conditions

The UHPLC‐QTOF‐MS analysis was performed on Waters Xevo G2‐XS QTof (Waters, USA). Chromatographic separations were conducted on a Waters Acquity UPLC BEH‐C18 (2.1 × 100 mm, 1.7 μm) chromatographic column (Lot: 186002352 Waters, USA) and the column temperature was programmed at 35°C (Zhang, He, et al. 2024). The mobile phases were 0.1% formic acid in water (A)‐acetonitrile (B), and the gradient elution conditions were as follows: 0–23 min, 5%–95% B; 23–26 min, 95% B; 26–26.01 min, 95%–5% B; 26.01–30 min, 5% B. The injection volume was 2 μL. Waters mass spectrometry adopted ESI+ and MS^E^ methods in which the data acquisition rate was 0.2 s; The range of *m*/*z* was 100–1500; collision gas was high purity argon, and the real‐time mass axis calibration solution (lock mass) was Leucine Enkephalin (LE) at a concentration of 200 ng/mL. In addition, capillary: 3.0 kV; sampling cone: 40 V; source offset: 80 V; desolvation temperatures: 450°C; desolvation gas: 900 L/h; collision energy: 10–40 V; as well as source temperatures: 120°C. Before sample analysis, the calibration of the mass axis and lock mass were performed.

### 2.5 Data Processing and Identification Algorithm Flow

The mass spectrometry of CRP, XCRP, and blank solvent was processed by Progenesis QI with the parameters as follows: Type of machine: high resolution; Polarity: Positive; Peak picking limits: automatic; Retention time: 1.00–26.00 min. Based on the above parameters, we obtained the quantized data of each sample, including retention time (Rt), mass‐to‐charge ratio (*m*/*z*), and ionic strength (*I*) (Wang, Zhang, He, Fu, et al. 2024; Wang, Zhang, Jing, Li, et al. 2024). Further, the obtained quantized data was saved as a “CSV file” in the format of digital matrix.

The algorithm flow was implemented by java language and can be divided into the following steps (Wang, Zhang, He, Fu, et al. 2024; Hou et al. 2023):

1. Blank deduction: to remove interfering ions originating from blank substrates in all samples, which could strengthen the match accuracy. The above algorithm would judge whether the ions in samples come from blank substrates and delete them using the following criteria: ions in samples and blank substrates had similar Rt and *m*/*z* (ΔRt ≤ 0.05 min and Δ*m*/*z* ≤ 0.05 Da) (Zhang, He, et al. 2024).
2. “Digital identity card” acquisition: firstly, The shared ions were extracted from the mass spectra of different batches of CRP from same region and were removed repetitively similar ions as “ion characterization” of CRP in this region in which the shared ions and similar ions had similar Rt and *m*/*z* (ΔRt ≤ 0.05 min and Δ*m*/*z* ≤ 0.05 Da). Then the collection of unique ions of each origin relative to other origins was screened out from each origin by comparison between the respective shared ions or between the shared ions and raw data. Finally the top N ions were retained as “digital identity card” for CRP from each origin, respectively based on the ordering of ionic strengths (Wang, Zhang, Jing, Li, et al. 2024; Hou et al. 2023).
3. Matching identification: in order to realize origin traceability analysis, the “digital identity card” of CRP from different regions was used as a benchmark for matching identification of CRP to feedback the matching credibility (MC). In this process, the criteria for matching identification were as follows: the ions in the “digital identity card” and samples to be identified had similar Rt and m/z. The formula of MC is as follows (Zhang, He, et al. 2024; Hou et al. 2023):

(1) $$\mathrm{MC}=\frac{\text{number}\;\text{of}\;\text{matched}\;\text{ions}}{\begin{array}{cccc} \text{number} & \text{of} & \text{ions} & \begin{array}{ccc} \text{in} & \text{digital} & \text{identity} \end{array} \end{array}}\times 100\%$$

4 Origin traceability analysis of CRP: on the basis of above method (1–3), the CRPs from different origin were analyzed by UHPLC‐QTOF‐MS/MS and used to extract “digital identity card” of CRP, meanwhile, 6 batches of XCRP in Table 1 were used to extract “digital identity card” of XCRP (geoauthentic herb) where the unique ions were compared between the respective shared ions. Then, the “digital identity cards” of CRP and XCRP were used as a benchmark for adulteration analysis to provide feedback on the matching credibility (MC).
5 Adulteration analysis: on the basis of above method (1–3), the 8 reference samples were analyzed by UHPLC‐QTOF‐MS/MS and used to extract “digital identity card” of CRP, meanwhile, 6 batches of XCRP in Table 1 were used to extract “digital identity card” of XCRP (geoauthentic herb) where the unique ions were compared between the shared ions and raw data. Then the “digital identity cards” of CRP and XCRP were used as a benchmark for adulteration analysis to provide feedback on the matching credibility (MC).

**TABLE 1:** The number of [Rt − *m*/*z* − *I*] units in CRPs of different regions.

Herbs	Origin	Batch	Number	Herbs	Origin	Batch	Number
CRP	Xinhui	XH01	3080	CRP	Hubei	HB01	3883
	Xinhui	XH02	3400		Hubei	HB02	3951
	Xinhui	XH04	3786		Hubei	HB04	4805
	Xinhui	XH05	4104		Hubei	HB05	5115
	Xinhui	XH07	3911		Guangxi	GX01	4327
	Xinhui	XH08	3894		Guangxi	GX02	4157
	Hunan	HN01	5569		Guangxi	GX04	3786
	Hunan	HN02	5111		Guangxi	GX05	5189
	Hunan	HN04	4743		Yunnan	YN01	3714
	Hunan	HN05	5986		Yunnan	YN02	3597
	Yunnan	YN04	4159		Yunnan	YN05	4434

## 3 Results

### 3.1 UHPLC‐QTOF‐MS Analysis

**FIGURE 1:**
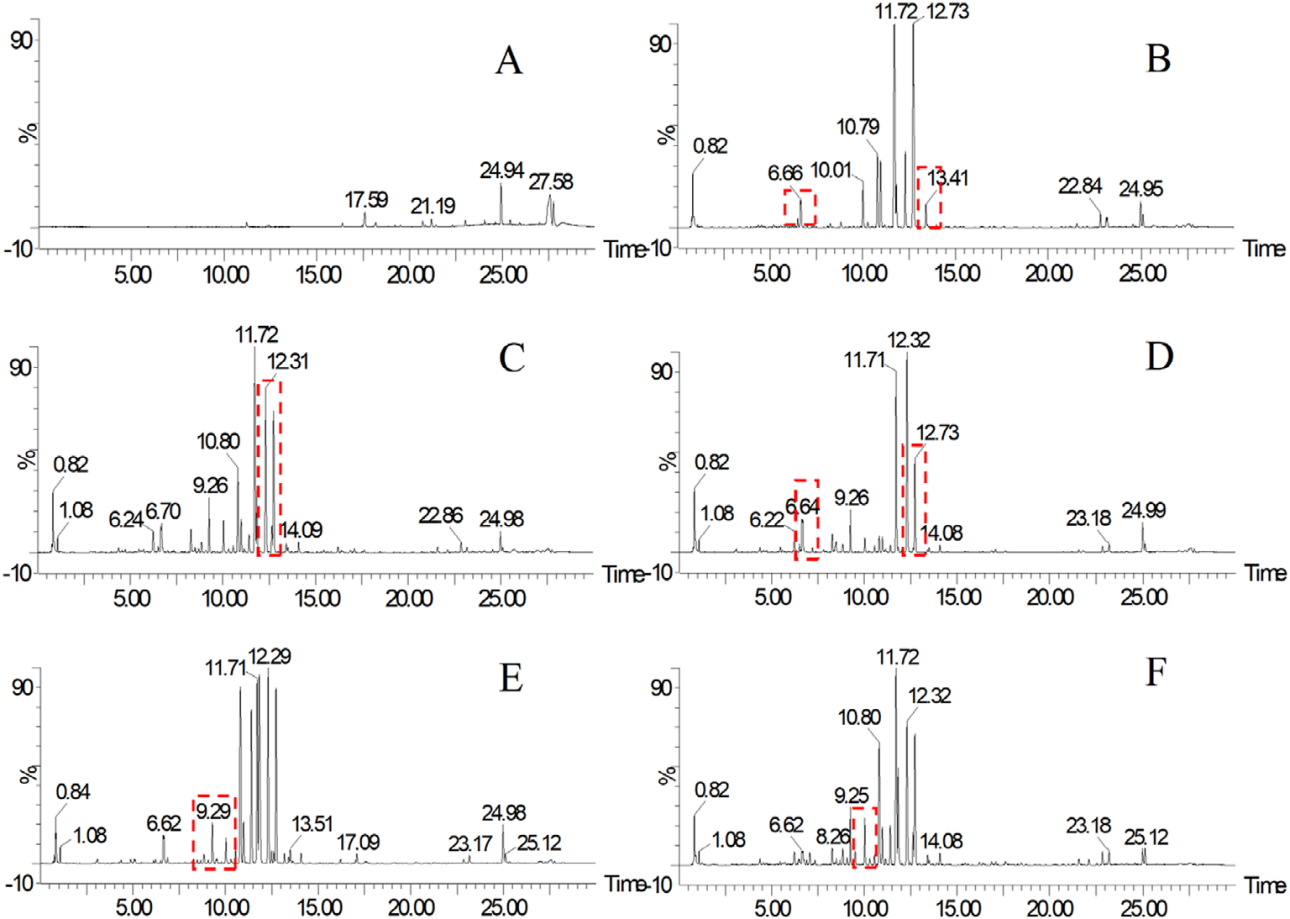
The base‐peak chromatogram of blank and CRP in different regions (A: blank; B: Xinhui; C: Hubei; D: Yunnan; E: Guangxi; F: Hunan; The base ions in the red border are the differential ion peaks).

Under the sample processing and experimental conditions, we obtained the base‐peak chromatogram of CRP from different regions. The base‐peak chromatogram of the blank and some samples is shown in Figure 1.

As shown in Figure 1, CRPs in different regions showed similar base‐peak chromatograms under the same detection conditions, which means that the difference in the base‐peak chromatograms of CRP from different places was not obvious, so it was very difficult to distinguish the origin of CRP only by the base‐peak chromatograms, and it needs further digital identification analysis to realize the study of origin traceability and adulteration.

### 3.2 Digital Processing

In this paper, the mass spectrometry information of methanol solvent was taken as “blank,” and they contain 3429–4464 [Rt − *m*/*z* − *I*] units respectively after being converted into data matrix by Progenesis QI. The mean value is 4022 with RSD = 9.75%. For CRPs of different regions that were used to extract “digital identity cards,” the number of [Rt − *m*/*z* − *I*] units of CRP was shown in the Table 1.

As shown in Table 1, the number of [Rt − *m*/*z* − *I*] of XCRP was all exceeded 3000, in which the XCRP with batch number XH05 had 4104 [Rt − *m*/*z* − *I*] units, meanwhile there were only 3080 [Rt − *m*/*z* − *I*] units in the XCRP with batch number XH01. In addition, the number of [Rt − *m*/*z* − *I*] of Hubei and Guangxi's CRPs was all exceeded 3700, in which the CRP with batch number GX05 had 5189 [Rt − *m*/*z* − *I*] units, but there were only 3883 [Rt − *m*/*z* − *I*] units in the CRP with batch number HB01. The above results showed that there were some differences in the number of [Rt − *m*/*z* − *I*] units in different batches of CRP in the same regions or CRP in different regions. It also illustrated from the side that the mass spectrometry information of samples from different batches or regions of CRP was different, and there were different chemical components at the molecular level that were caused by different producing areas, storage time, and individual differences, which were in line with the actual situation. On the other hand, there must inevitably be differences between the same medicinal materials from different places or different batches of the same medicinal materials from the same place, which also suggests that we must comprehensively consider different batches, different years or different regions of CRP to obtain accurate “digital identity card” of CRP and XCRP.

In addition, the differences between different batches of the same herbs did not affect this research. This research mainly relied on the digital representation of chemical components by mass spectrometry at the molecular level to extract shared data as core digital matrices and construct the “digital identity card” of CRPs. The research idea focused on the common chemical components of different batches (or different regions) of the same herbs, rather than the different components. In addition, the digital characterization of mass spectrometry information was used instead of identifying compounds, which can greatly improve the efficiency of analysis through digital matching and comparison (Zhang, He, et al. 2024).

### 3.3 “Digital Identity Card” Acquisition of CRP and XCRP

Based on the digitalization of mass spectrometry, different batches of CRPs from different producing areas in Table 1 were used to extract their shared data as “digital identity cards” respectively. After extracting the common data, the interfering ions existing simultaneously in the methanol solvents and samples were removed. During the above steps of de‐blanking and extracting shared data, the parameter thresholds of retention time (Rt) and mass‐to‐charge ratio (*m*/*z*) were set in bundles as ΔRt ≤ 0.05 min and Δ*m*/*z* ≤ 0.05 Da. That is to say, all mass spectrometry data that satisfy the threshold deviation were constructed in the new data matrix. Further, the top 100 [Rt − *m*/*z* − *I*] data matrices were screened as “digital identity cards” of CRP in different regions, respectively, based on the ionic strength ordering. The above processing flow was all realized by using the JAVA programming language.

In the “digital identity card” acquisition of CRP in different regions, we investigated different retention time (Rt) and mass‐to‐charge ratio (*m*/*z*)thresholds. Originally, we set the deviation threshold of retention time (Rt) and mass‐to‐charge ratio (*m*/*z*) as ΔRt ≤ 0.05 min and Δ*m*/*z* ≤ 0 Da, respectively. However, the shared ions cannot be obtained under these harsh conditions. Further, the Rt and *m*/*z* were set as ΔRt ≤ 0.05 min and Δ*m*/*z* ≤ 0.01 Da, the shared ions in Guangxi's and Yunnan's CRP were all less than 100, which did not allow us to make further feature screening according to ionic strength. When the Rt and *m*/*z* were set as ΔRt ≤ 0.05 min and Δ*m*/*z* ≤ 0.05 Da, the shared ions in CRP of different regions were all higher than 1000, which leaves us a certain processing space to make feature screening according to ionic strength. The above was mainly due to that the instrument state was different in different periods, and detecting different batches of CRP samples in different periods could indeed bring errors. In addition, the difference in the external environment can also bring deviation, especially the laboratory temperature. This also suggested that we must set a reasonable deviation threshold of Rt and *m*/*z* to extract shared data. To ensure the number of shared ions and the space for screening according to ion strength, based on referring to relevant literature (Hou et al. 2023; Li et al. 2023), we finally determined that the parameter thresholds were ΔRt ≤ 0.05 min and Δ*m*/*z* ≤ 0.05 Da. At the same time, the Rt did drift but did not exceed 0.05 min, so it remained unchanged at 0.05 min in the Rt deviation threshold setting.

### 3.4 Origin Traceability Analysis of CRP in Different Regions

Firstly, the samples of CRP in different regions that were used for origin traceability were processed by UHPLC‐QTOF‐MS to obtain mass spectrometry information, which was converted into quantized data matrices by Progenesis QI. Then the “digital identity card” of CRP in different regions as the benchmark to match them with the data matrices of CRP that are used for origin traceability sequentially to feed back the matching credibility (MC). The origin traceability analysis was realized according to the MC.

In the origin traceability analysis of CRP in different regions, the deviation threshold of Rt and m/z was set as ΔRt ≤ 0.05 min and Δ*m*/*z* ≤ 0.05 Da. Eleven batches of test samples, including 3 batches of XCRP, 2 batches of Hunan's CRP, 2 batches of Hubei's CRP, 2 batches of Guangxi's CRP, and 2 batches of Yunnan's CRP. The matching results of XCRP are shown in Table 2. As shown in Table 2, the MC of XCRP in different batches compared with the “digital identity card” of XCRP was all not less than 95.00%, in which the MC of XCRP in batch of XH03 and XH06 was all 100%, followed by the XCRP in batch of XH09 with MC = 95.00%. At the same time, the MC was not higher than 43.00% when the XCRP was compared with the “digital identity card” of Hunan's CRP, Hubei's CRP, Guangxi's CRP, and Yunnan's CRP. The MC of XCRP compared with its own “digital identity card” was at least 2.2 times that of XCRP compared with the “digital identity card” of CRP in the other four regions. Table 3 shows the matching results of Guangxi's CRP, and it can be seen that the MC of Guangxi's CRP with batch numbers GX03 and GX06 was 91.00% and 109.00%, respectively, when compared with the “digital identity card” of Guangxi's CRP itself. Meanwhile, compared with the “digital identity card” of CRP in the other four regions, Guangxi's CRP with batch GX03 had a maximum MC = 39.00% when matched with the “digital identity card” of Yunnan's CRP. It was worth noting that the reason why the MC was greater than 100% was due to the existence of a deviation threshold, which led to the phenomenon that one ion in a “digital identity card” can match multiple sample ions within the set threshold deviation range. For example, both *m*/*z* 258.08 [M + H]^+^ and *m*/z 258.18 [M + 2H]^+^ in sample GX06 can be matched to *m*/*z* 258.13 in the “digital identity card” of Guangxi's CRP. Table 4 shows the matching results of Hunan's CRP. The MC of Hunan's CRP compared with its “digital identity card” was not less than 92.00% and the MC of Hunan's CRP compared with CRP's “digital identity card” in the other four regions was not higher than 42.00%, which is a gap of more than 2 times in MC. In addition, as shown in Tables S2 and S3, the MCs of Hubei's CRP and Yunnan's CRP compared with their own “digital identity card” were all not less than 90.00%. But compared with non‐self CRP's “digital identity card,” the MCs of Hubei's CRP and Yunnan's CRP were all not higher than 43.00%, which was far lower than the MC when compared with their own “digital identity card.”

**TABLE 2:** The matching credibility results of XCRP.

Herbs	Batches	Match ions	Ions in digital identity card	MC (%)
XCRP	XH03	100	100‐XCRP	100.00
	XH06	100	100‐XCRP	100.00
	XH09	95	100‐XCRP	95.00
	XH03	11	100‐Hunan's CRP	11.00
	XH06	13	100‐Hunan's CRP	13.00
	XH09	16	100‐Hunan's CRP	16.00
	XH03	29	100‐Hubei's CRP	29.00
	XH06	24	100‐Hubei's CRP	24.00
	XH09	24	100‐Hubei's CRP	24.00
	XH03	43	100‐Guangxi's CRP	43.00
	XH06	25	100‐Guangxi's CRP	25.00
	XH09	39	100‐Guangxi's CRP	39.00
	XH03	22	100‐Yunnan's CRP	22.00
	XH06	17	100‐Yunnan's CRP	17.00
	XH09	21	100‐Yunnan's CRP	21.00

**TABLE 3:** The matching credibility results of Guangxi's CRP.

Herbs	Batches	Match ions	Ions in digital identity card	MC (%)
Guangxi's CRP	GX03	91	100‐Guangxi's CRP	91.00
	GX06	109	100‐Guangxi's CRP	109.00
	GX03	18	100‐XCRP	18.00
	GX06	25	100‐XCRP	25.00
	GX03	20	100‐Hunan's CRP	20.00
	GX06	16	100‐Hunan's CRP	16.00
	GX03	24	100‐Hubei's CRP	24.00
	GX06	26	100‐Hubei's CRP	26.00
	GX03	39	100‐Yunnan's CRP	39.00
	GX06	33	100‐Yunnan's CRP	33.00

**TABLE 4:** The matching credibility results of Hunan's CRP.

Herbs	Batches	Match ions	Ions in digital identity card	MC (%)
Hunan's CRP	HN03	92	100‐Hunan's CRP	92.00
	HN06	98	100‐Hunan's CRP	98.00
	HN03	37	100‐XCRP	37.00
	HN06	39	100‐XCRP	39.00
	HN03	30	100‐Guangxi's CRP	30.00
	HN06	30	100‐Guangxi's CRP	30.00
	HN03	38	100‐Hubei's CRP	38.00
	HN06	42	100‐Hubei's CRP	42.00
	HN03	32	100‐Yunnan's CRP	32.00
	HN06	41	100‐Yunnan's CRP	41.00

The above results showed that there was a significant difference in the MC obtained through matching sample data with the “digital identity card” of CRP from different regions. In short, the MCs were not less than 90% when compared to their own “digital identity card” meanwhile the MCs were not more than 43% when compared to non‐self “digital identity card,” in which the MCs varied by 47 points and more than one times. It also further illustrated that the “digital identity card” of CRPs from different origins had certain specificity. According to the “digital identity card” of CRPs from different origins, it can realized traceability analysis of CRPs.

In the process of analysis, the matches when the top 100, 150, and 200 [Rt − *m*/*z* − *I*] were output as “digital identity cards” of CRP from different origins, according to the ionic strength ordering, were explored. The results illustrated that the MC showed a slight downward trend with the increase of ion number. For example, the MC of XH03 was 98.66% when compared with XCRP's “digital identity card” that contained the top 150 [Rt − *m*/*z* − *I*], and the MC = 97.00% when compared with XCRP's “digital identity card” that contains the top 200 [Rt − *m*/*z* − *I*]. The MC of HB06 was 85.33% when the “digital identity card” of Hubei's CRP contained the top 150 [Rt − *m*/*z* − *I*], and the MC = 84.00% when the “digital identity card” of Hubei's CRP contained the top 200 [Rt − *m*/*z* − *I*]. All the above were lower than the MC in Tables 2 and S2. Finally, the top 100 [Rt − *m*/*z* − *I*] were outputted as “digital identity cards” of CRPs from different origins.

### 3.5 Identification Analysis of Adulteration

**FIGURE 2:**
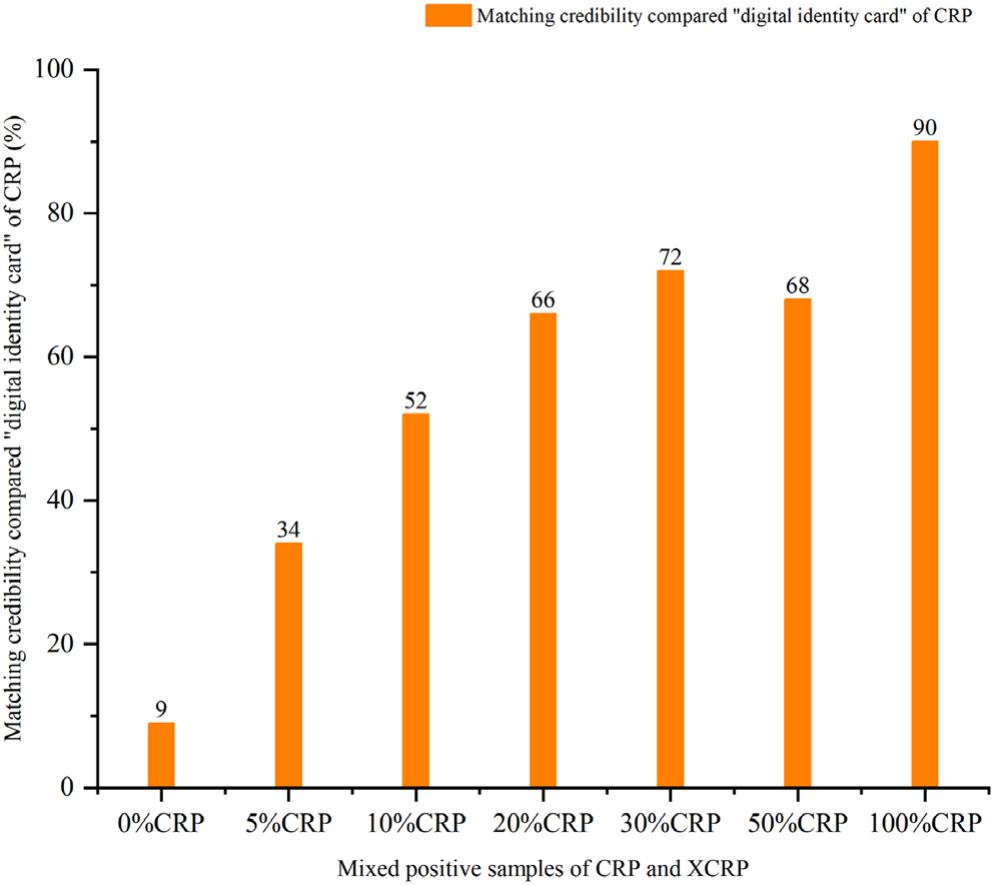
The matching results of mixed positive samples with different proportions of CRP compared with “digital identity card” of CRP.

**FIGURE 3:**
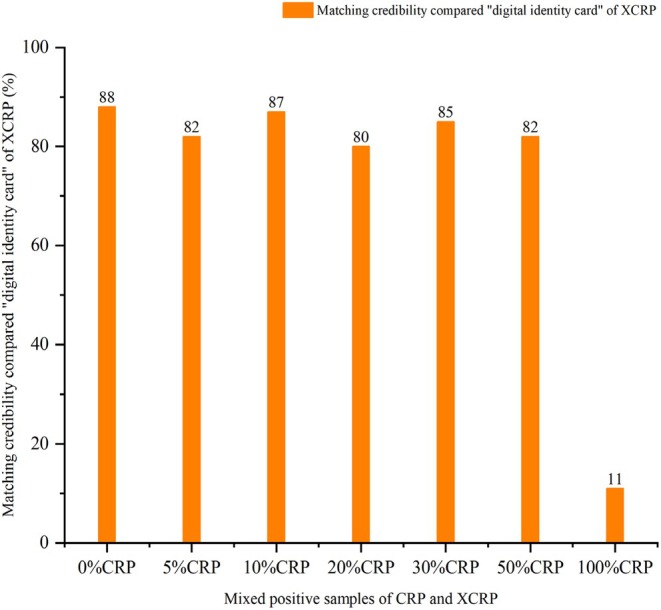
The matching results of mixed positive samples with different proportions of CRP compared with “digital identity card” of XCRP.

In the identification analysis of adulteration, the top 100 [Rt − *m*/*z* − *I*] were also outputted as “digital identity cards” of XCRP and reference samples. Taking the “digital identity cards” of XCRP and reference sample (CRP) as the benchmark to match mixed positive samples. the deviation threshold of Rt and *m*/*z* was ΔRt ≤ 0.05 min and Δ*m*/*z* ≤ 0.05 Da. The matching results are shown in Figures 2 and 3.

As illustrated in Figure 2, with the increasing proportion of CRP in the mixed sample, the MC between the mixed sample and the “digital identity card” of CRP was getting higher, and it showed an upward trend on the whole. When there was no CRP in the sample, the MC between XCRP and the “digital identity card” of CRP was only 9%. Meanwhile, the MC = 34% between the mixed sample with 5% CRP and CRP “digital identity card,” which was more than 3.7 times that of “0% CRP.” From the mixed sample with 10% CRP to the mixed sample with 50% CRP, the MCs were all greater than 50%, in which the highest MC of 30% CRP was 72%. When there was no XCRP in the sample, the MC between 100% CRP with CRP “digital identity card” was up to 90%. At the same time, the matching results of samples compared with the “digital identity card” of XCRP were shown in Figure 3, from the sample with 0%CRP to the sample with 50%CRP, the MCs were all greater than 80%. In addition, the MC between 100% CRP with a “digital identity card” of XCRP was only 11%. Based on the comprehensive analysis of Figures 2 and 3, based on the “digital identity card” of CRP and XCRP, even if only 5% CRP was added to the mixed sample, it can still be effectively detected according to the matching credibility. It also illustrated that the “digital identity card” of XCRP and CRP had certain specificity and can realize the identification analysis of adulteration of CRP. Through identification analysis of adulteration and considering the deviation threshold and fluctuation of detection data, we suggested that the MC threshold may be set to 50%, that is to say, when the unknown sample is matched with the “digital identity card” of CRP, the MC greater than 50% is deemed to be detected.

## 4 Discussion

### 4.1 Discussion of UHPLC‐QTOF‐MS Experimental Conditions

The UHPLC‐QTOF‐MS was widely used in the analysis field of plant herbs, which can provide us with massive data information reflecting chemical components (Lee et al. 2021; Wang, Wu, Li, Guo, et al. 2024). In this paper, we also explored the sample pretreatment methods, such as extraction solvent methanol and water under ultrasound or heating reflux. The results showed that the mass spectrometry data obtained when methanol was used as the extraction solvent were significantly higher than those of water; moreover, there was essentially no difference between treatments of ultrasonic extraction and heat reflux (Zhang, He, et al. 2024). So we finally chose ultrasonic extraction (power: 500 W, frequency: 40 kHz) with methanol as the solvent for 30 min. In addition, we used the MSE data‐independent acquisition mode to obtain the parent ions and secondary fragment ions at the same time, thus ensuring more data information (Wang, Wu, Li, Guo, et al. 2024). Moreover, we investigated the mass spectrum information of CRPs when the collision energies were 10–30, 10–40, and 10–50 V, respectively. It was distinct that the mass spectrum had the most abundant data information with the collision energy being 10–40 V (Zhang, He, et al. 2024; Wang, Wu, Li, Guo, et al. 2024).

As for sample treatment, to better extract the chemical components in CRP and XCRP, the samples were crushed into powder and filtered by a No. 3 sieve. On the market, the main products are intact CRP and XCRP. However, the adulteration ratio of intact samples is difficult to be accurate. Therefore, in adulteration analysis, sample powder is used to prepare positive samples with an accurate adulteration ratio. The adulteration analysis using sample powder does not affect the analysis results. Even the UHPLC‐QTOF‐MS analysis of intact CRP needs to be crushed and extracted.

### 4.2 Discussion on “Digital Identity Card”

In traceability analysis, the acquisition of a “digital identity card” was based on samples from different batches within the same origin to extract the shared data, which ensured maximum control of differences in CRP of the same origin. Then, the collection of unique ions of each origin relative to other origins was screened out from each origin by comparison between the respective shared ions from different batches within the same origin. The above practice also took into full consideration the differences between CRPs from different origins. At the same time, in adulteration analysis, we classified XCRP as a geoauthentic herb and divided Guangxi's CRP, Hunan's CRP, Hubei's CRP, and Yunnan's CRP into non‐geoauthentic herbs. Further, the CRPs were mixed to make 8 reference samples, which were used to extract the “digital identity card” of the CRP. This ensured that the data from both CRP and XCRP were balanced and less likely to produce false positives, and took into account the CRPs from different producing areas, which improved the applicability and accuracy. In addition, matching comparisons between shared ions based on CRP from different origins cannot meet the needs of adulteration analysis when extracting a “digital identity card.” For example, the MC = 47% when the 0% CRP (XCRP) was compared with the “digital identity card” of CRP, and the MC = 66% when the 5%CRP was compared with the “digital identity card” of CRP in which there was a difference, but not significant, between MCs. So we changed our strategy by sequentially comparing the shared ions of CRP with the original data used to extract the “digital identity card” of XCRP and deleting the common ions to obtain the “digital identity card” of CRP. Similarly, the above method was used to obtain the “digital identity card” of XCRP. This approach can greatly increase the difference between MCs. For example, the MC = 9% when the 0% CRP (XCRP) was compared with the “digital identity card” of CRP and the MC = 34% when the 5% CRP was compared with the “digital identity card” of CRP in which there was a significant difference between MCs. At the same time, the MC = 11% when the 100% CRP was compared with the “digital identity card” of XCRP. So we used this method to extract the “digital identity card” for adulteration analysis.

On the other hand, compared with previous studies (Wang, Zhang, He, Fu, et al. 2024; Hou et al. 2023), the “digital identity card” is not only innovative in concept but also in the algorithm. “Digital identity card” is no longer fixed and limited ions but can be dynamically updated with the increase of sample batches, so it is more suitable for practical analysis. The limit of 1.0 × 10^4^ for ion intensity has been removed, ensuring more ion data information. The elimination algorithm step for comparing the shared ion data with the original data was added, thus ensuring better specificity. The deviation threshold and ion output window are retained, allowing optimization of Rt, *m*/*z* deviation, and the number of ions output according to analytical needs, making analysis more convenient. Finally, the algorithm implementation of “digital identity card” adopts a multi‐threaded design, which can greatly save the matching and comparison time and improve the identification efficiency. Moreover, compared with the previous chemical composition adulteration analysis (Chen et al. 2024), the “digital identity card” realizes the identification of CRP adulterated XCRP based on the rational and effective utilization of unknown chemical compositions for the first time. It can identify CRP in XCRP efficiently and accurately.

### 4.3 Digital Identification of Plant Medicine

With the advent of the digital information era of Chinese medicine, identification analysis of plant medicine can quickly realize the digital identification at the individual level, which is conducive to the breakthrough of rapid detection technology (Zhang et al. 2023; Zhu et al. 2023; Nair 2022; Cao et al. 2021). In this paper, the “digital identity card” of CRP and XCRP had a certain specificity and representation, which can provide a reference for the establishment of the “digital identity card” of plant medicine and provides a way to realize digital identification of plant medicine. The advantage of digital identification based on a “digital identity card” is that it does not need to identify the specific compound structure and molecular formula, and can incorporate all the obtained quantized data into the digital matrix to digitally characterize the individual plant medicine. The method focuses on the identification of herbs at the individual level and is no longer limited to a few compounds with a clear structure and known molecular formula. At the same time, it can make full use of the quantized information of unknown components to make identification analysis more reasonable and reliable.

### 4.4 Research Limitations and Perspectives

Admittedly, this study also has some limitations. The 33 batches of samples used in this study were commissioned to NIFDC for collection. The sample size is relatively small and does not fully cover the sample variation. Further samples need to be collected for subsequent analysis. Fortunately, the “digital identity card” algorithm can expand the samples at any time. Moreover, it focuses on the common chemical components of CRPs in the same origin rather than the differential chemical components, and the different chemical components of CRPs in different origins. Therefore, it can control the influence of sample differences to some extent. In addition, methodological research can be carried out based on the “digital identity card” to establish a scientific identification methodology system to help CRP quality supervision.

## Author Contributions

**Xianrui Wang:** data curation (equal), methodology (equal), writing – original draft (equal). **Jiating Zhang:** writing – original draft (equal). **Fangliang He:** data curation (equal). **Wenguang Jing:** conceptualization (equal), data curation (equal). **Xiaohan Guo:** data curation (equal), software (equal). **Minghua Li:** data curation (equal), investigation (equal). **Kunzi Yu:** data curation (equal), methodology (equal), software (equal), writing – review and editing (equal). **Jianbo Yang:** data curation (equal), methodology (equal), software (equal), validation (equal). **Xianlong Cheng:** funding acquisition (equal), project administration (equal), supervision (equal). **Feng Wei:** funding acquisition (equal), project administration (equal), supervision (equal).

## Conflicts of Interest

The authors declare no conflicts of interest.

## Supporting information


Data S1.

## Data Availability

The data that support the findings of this study are available on request from the corresponding author.
