# Supplementary material for: Traceability and Adulteration Analysis of Citri Reticulatae Pericarpium Based on “Digital Identity Card” and UHPLC‐QTOF‐MS Analysis
Source: Food Sci Nutr. 2025 Apr 30;13(5):e70163. doi: 10.1002/fsn3.70163 (PMC12041654; doi:10.1002/fsn3.70163)
Supplement: Supplementary file 1 — Data S1. [file FSN3-13-e70163-s001.docx]

Table S1 The detailed information of experiment samples

| Herbs | Batch Number | Years | Material Source | Application | Production area |
| --- | --- | --- | --- | --- | --- |
| Citri Reticulatae Pericarpium | HN01 | 5 | National Institutes for Food and Drug Control | Extract "digital identity" | Hunan, China |
| Citri Reticulatae Pericarpium | HN02 | 5 | National Institutes for Food and Drug Control | Extract "digital identity" | Hunan, China |
| Citri Reticulatae Pericarpium | HN03 | 7 | National Institutes for Food and Drug Control | External Verification | Hunan, China |
| Citri Reticulatae Pericarpium | HN04 | 3 | National Institutes for Food and Drug Control | Extract "digital identity" | Hunan, China |
| Citri Reticulatae Pericarpium | HN05 | 6 | National Institutes for Food and Drug Control | Extract "digital identity" | Hunan, China |
| Citri Reticulatae Pericarpium | HN06 | 10 | National Institutes for Food and Drug Control | External Verification | Hunan, China |
| Citri Reticulatae Pericarpium | HB01 | 4 | National Institutes for Food and Drug Control | Extract "digital identity" | Hubei, China |
| Citri Reticulatae Pericarpium | HB02 | 5 | National Institutes for Food and Drug Control | Extract "digital identity" | Hubei, China |
| Citri Reticulatae Pericarpium | HB03 | 5 | National Institutes for Food and Drug Control | External Verification | Hubei, China |
| Citri Reticulatae Pericarpium | HB04 | 10 | National Institutes for Food and Drug Control | Extract "digital identity" | Hubei, China |
| Citri Reticulatae Pericarpium | HB05 | 10 | National Institutes for Food and Drug Control | Extract "digital identity" | Hubei, China |
| Citri Reticulatae Pericarpium | HB06 | 15 | National Institutes for Food and Drug Control | External Verification | Hubei, China |
| Citri Reticulatae Pericarpium | GX01 | 8 | National Institutes for Food and Drug Control | Extract "digital identity" | Guangxi, China |
| Citri Reticulatae Pericarpium | GX02 | 7 | National Institutes for Food and Drug Control | Extract "digital identity" | Guangxi, China |
| Citri Reticulatae Pericarpium | GX03 | 5 | National Institutes for Food and Drug Control | External Verification | Guangxi, China |
| Citri Reticulatae Pericarpium | GX04 | 5 | National Institutes for Food and Drug Control | Extract "digital identity" | Guangxi, China |
| Citri Reticulatae Pericarpium | GX05 | 10 | National Institutes for Food and Drug Control | Extract "digital identity" | Guangxi, China |
| Citri Reticulatae Pericarpium | GX06 | 3 | National Institutes for Food and Drug Control | External Verification | Guangxi, China |
| Citri Reticulatae Pericarpium | YN01 | 5 | National Institutes for Food and Drug Control | Extract "digital identity" | Yunnan, China |
| Citri Reticulatae Pericarpium | YN02 | 5 | National Institutes for Food and Drug Control | Extract "digital identity" | Yunnan, China |
| Citri Reticulatae Pericarpium | YN03 | 6 | National Institutes for Food and Drug Control | External Verification | Yunnan, China |
| Citri Reticulatae Pericarpium | YN04 | 10 | National Institutes for Food and Drug Control | Extract "digital identity" | Yunnan, China |
| Citri Reticulatae Pericarpium | YN05 | 12 | National Institutes for Food and Drug Control | Extract "digital identity" | Yunnan, China |
| Citri Reticulatae Pericarpium | YN06 | 3 | National Institutes for Food and Drug Control | External Verification | Yunnan, China |
| Citri Reticulatae Pericarpium | XH01 | 3 | National Institutes for Food and Drug Control | Extract "digital identity" | Xinhui, China |
| Citri Reticulatae Pericarpium | XH02 | 5 | National Institutes for Food and Drug Control | Extract "digital identity" | Xinhui, China |
| Citri Reticulatae Pericarpium | XH03 | 10 | National Institutes for Food and Drug Control | External Verification | Xinhui, China |
| Citri Reticulatae Pericarpium | XH04 | 10 | National Institutes for Food and Drug Control | Extract "digital identity" | Xinhui, China |
| Citri Reticulatae Pericarpium | XH05 | 15 | National Institutes for Food and Drug Control | Extract "digital identity" | Xinhui, China |
| Citri Reticulatae Pericarpium | XH06 | 15 | National Institutes for Food and Drug Control | External Verification | Xinhui, China |
| Citri Reticulatae Pericarpium | XH07 | 8 | National Institutes for Food and Drug Control | Extract "digital identity" | Xinhui, China |
| Citri Reticulatae Pericarpium | XH08 | 6 | National Institutes for Food and Drug Control | Extract "digital identity" | Xinhui, China |
| Citri Reticulatae Pericarpium | XH09 | 7 | National Institutes for Food and Drug Control | External Verification | Xinhui, China |

Table S2 The matching credibility results of Hubei’s CRP

| Herbs | Batches | Match ions | Ions in digital identity card | MC(%) |
| --- | --- | --- | --- | --- |
| Hubei’s  CRP | HB03 | 96 | 100-Hubei’s CRP | 96.00 |
|  | HB06 | 90 | 100-Hubei’s CRP | 90.00 |
|  | HB03 | 33 | 100-XCRP | 33.00 |
|  | HB06 | 31 | 100-XCRP | 31.00 |
|  | HB03 | 16 | 100-Guangxi’s CRP | 16.00 |
|  | HB06 | 28 | 100-Guangxi’s CRP | 28.00 |
|  | HB03 | 28 | 100-Hunan’s CRP | 28.00 |
|  | HB06 | 39 | 100-Hunan’s CRP | 39.00 |
|  | HB03 | 36 | 100-Yunnan’s CRP | 36.00 |
|  | HB06 | 43 | 100-Yunnan’s CRP | 43.00 |

Table S3 The matching credibility results of Yunnan’s CRP

| Herbs | Batches | Match ions | Ions in digital identity card | MC(%) |
| --- | --- | --- | --- | --- |
| Yunnan’s  CRP | YN03 | 93 | 100-Yunnan’s CRP | 93.00 |
|  | YN06 | 90 | 100-Yunnan’s CRP | 90.00 |
|  | YN03 | 19 | 100-XCRP | 19.00 |
|  | YN06 | 18 | 100-XCRP | 18.00 |
|  | YN03 | 17 | 100-Guangxi’s CRP | 17.00 |
|  | YN06 | 15 | 100-Guangxi’s CRP | 15.00 |
|  | YN03 | 10 | 100-Hunan’s CRP | 10.00 |
|  | YN06 | 20 | 100-Hunan’s CRP | 20.00 |
|  | YN03 | 24 | 100-Hubei’s CRP | 24.00 |
|  | YN06 | 31 | 100-Hubei’s CRP | 31.00 |
